# Imaging features of the PI-RADS for predicting extraprostatic extension of prostate cancer: systematic review and meta-analysis

**DOI:** 10.1186/s13244-023-01422-9

**Published:** 2023-05-08

**Authors:** Moon Hyung Choi, Dong Hwan Kim, Young Joon Lee, Sung Eun Rha, Ji Youl Lee

**Affiliations:** 1 Department of Radiology, Eunpyeong St. Mary’s Hospital, College of Medicine, The Catholic University of Korea, Seoul, Republic of Korea; 2 Department of Radiology, Seoul St. Mary’s Hospital, College of Medicine, The Catholic University of Korea, 222 Banpo-daero, Seocho-gu, Seoul, 06591 Republic of Korea; 3 Department of Urology, Seoul St. Mary’s Hospital, College of Medicine, The Catholic University of Korea, Seoul, Republic of Korea

**Keywords:** Prostatic neoplasms, Neoplasm staging, Magnetic resonance imaging, Systematic review, Meta-analysis

## Abstract

**Objectives:**

To systematically determine the diagnostic performance of each MRI feature of the PI-RADS for predicting extraprostatic extension (EPE) in prostate cancer.

**Methods:**

A literature search in the MEDLINE and EMBASE databases was conducted to identify original studies reporting the accuracy of each feature on MRI for the dichotomous diagnosis of EPE. The meta-analytic pooled diagnostic odds ratio (DOR), sensitivity, specificity, and their 95% confidence intervals (CIs) were obtained using a bivariate random-effects model.

**Results:**

After screening 1955 studies, 17 studies with a total of 3062 men were included. All six imaging features, i.e., bulging prostatic contour, irregular or spiculated margin, asymmetry or invasion of neurovascular bundle, obliteration of rectoprostatic angle, tumor-capsule interface > 10 mm, and breach of the capsule with evidence of direct tumor extension, were significantly associated with EPE. Breach of the capsule with direct tumor extension demonstrated the highest pooled DOR (15.6, 95% CI [7.7–31.5]) followed by tumor-capsule interface > 10 mm (10.5 [5.4–20.2]), asymmetry or invasion of neurovascular bundle (7.6 [3.8–15.2]), and obliteration of rectoprostatic angle (6.1 [3.8–9.8]). Irregular or spiculated margin showed the lowest pooled DOR (2.3 [1.3–4.2]). Breach of the capsule with direct tumor extension and tumor-capsule interface > 10 mm showed the highest pooled specificity (98.0% [96.2–99.0]) and sensitivity (86.3% [70.0–94.4]), respectively.

**Conclusions:**

Among the six MRI features of prostate cancer, breach of the capsule with direct tumor extension and tumor-capsule interface > 10 mm were the most predictive of EPE with the highest specificity and sensitivity, respectively.

**Graphical abstract:**

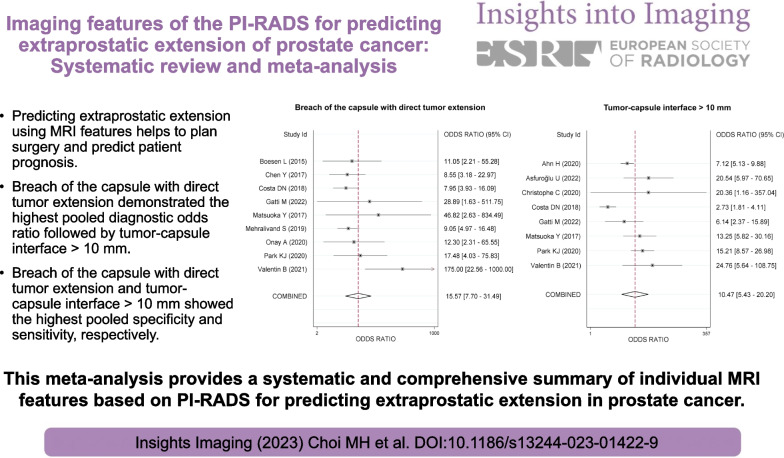

**Supplementary Information:**

The online version contains supplementary material available at 10.1186/s13244-023-01422-9.

## Abstract (highlights)

**Clinical relevance statement:**

This meta-analysis provides a systematic and comprehensive summary of individual MRI features based on PI-RADS for predicting extraprostatic extension in prostate cancer.

**Key points:**

1. Predicting extraprostatic extension using MRI features helps to plan surgery and predict patient prognosis.
2. Breach of the capsule with direct tumor extension demonstrated the highest pooled diagnostic odds ratio followed by tumor-capsule interface > 10 mm.
3. Breach of the capsule with direct tumor extension and tumor-capsule interface > 10 mm showed the highest pooled specificity and sensitivity, respectively

**Supplementary Information:**

The online version contains supplementary material available at 10.1186/s13244-023-01422-9.

## Introduction

Prostate cancer (PCa), one of the most common malignancies in males worldwide [1, 2], exhibits a wide spectrum of tumor aggressiveness, leading to diverse prognoses [3, 4]. As a result, treatment choices for PCa differ according to the characteristics of the tumor [5]. Active surveillance without treatment can be used for clinically insignificant cancer, whereas focal therapy is available for organ-confined PCa. Even with many treatment options, surgery remains a radical treatment. Therefore, the extent of PCa is critical in determining the best treatment option and planning surgery.

Radiological examination, as well as clinical information and biopsy results, are important in understanding the characteristics of PCa. Prostate magnetic resonance imaging (MRI) is considered the most accurate imaging technique for evaluating the prostate gland due to its ability to depict the anatomy of the prostate gland well [6]. Moreover, the value of prebiopsy MRI has been proven, and the role of prostate MRI has been expanded to detection and risk classification prior to the pathologic confirmation of PCa [7–10]. Particularly the tumor region beyond the prostate boundary, known as extraprostatic extension (EPE), should be thoroughly evaluated. Positive EPE, which indicates that PCa is more aggressive, is important for risk categorization since EPE is associated with a higher risk of biochemical recurrence and metastasis after radical prostatectomy (RP) [11, 12].

According to the Prostate Imaging Reporting and Data System (PI-RADS), suspected clinically significant PCa with EPE is categorized as PI-RADS 5, indicating a high probability of clinically significant cancer [13]. The probability of EPE was scored with a five-point scale in PI-RADS version 1 [14]. However, in PI-RADS versions 2 and 2.1, the EPE scoring system was eliminated, and individual EPE-related findings (bulging prostatic contour, irregular or spiculated margin, asymmetry or invasion of the neurovascular bundle, obliteration of the rectoprostatic angle, tumor-capsule interface > 10 mm, and breach of the capsule with evidence of direct tumor extension) were described instead [13]. The diverse nature of these findings might lead to variations in the diagnostic performance of MRI in predicting EPE. Therefore a new EPE grading system incorporating imaging findings was proposed and validated [15, 16]. Although several meta-analyses have reported the overall performance of MRI for detecting EPE based on different scoring schemes, the reported results vary widely, and the MRI definitions for EPE are sometimes ambiguous [17–20]. In addition, no attempt has yet been made to generate a systematic summary of the performance of each imaging feature that may be more clinically relevant when interpreting MRI to determine EPE. Therefore, we aimed to systematically determine the diagnostic performance of each imaging feature of the PI-RADS in predicting EPE of PCa.

## Materials and methods

This systematic review and meta-analysis was conducted based on the Preferred Reporting Items for Systematic Review and Meta-Analysis of Diagnostic Test Accuracy guidelines [21]. The study protocol was registered in the PROSPERO international prospective register of systematic reviews (reference number: CRD42022355301). Two radiologists (each with ≥ 10 years of experience in prostate imaging) independently performed the literature search, study selection, data extraction, and study quality assessment, and any disagreements were resolved by consensus and confirmed by a third reviewer.

### Literature search strategy and study selection criteria

A literature search of the PubMed MEDLINE and EMBASE databases was conducted to identify original publications reporting the diagnostic performance of the MRI features of PI-RADS (version 2 or 2.1) for predicting EPE in PCa. The following search queries were used: prostat* AND (“magnetic resonance” OR MR OR MRI) AND (extracapsular OR extraprostatic). The literature search was conducted on June 15, 2022, without a limitation on the start date.

The inclusion criteria were as follows: (1) population: patients with suspected or diagnosed PCa; (2) index test: prostate MRI; (3) reference standard: histopathological results after RP; (4) outcomes: diagnostic performance of six MRI features as defined in PI-RADS version 2 or 2.1 (i.e., bulging prostatic contour, irregular or spiculated margin, asymmetry or invasion of the neurovascular bundle, obliteration of the rectoprostatic angle, tumor-capsule interface > 10 mm, and breach of the capsule with evidence of direct tumor extension) for EPE of PCa; and (5) study design: observational studies (prospective or retrospective) and clinical trials. The exclusion criteria included the following: (1) case reports, letters, review articles, editorials, scientific abstracts, systematic reviews, and meta-analyses; (2) non-English articles; (3) studies focusing on topics other than the area of interest of this study (e.g., overall local staging of prostate MRI or diagnostic performance of MRI features not included in PI-RADS); (4) studies with insufficient data to construct a diagnostic 2-by-2 table between imaging tests and the reference standard diagnosis of EPE; and (5) studies that used suboptimal technical parameters of MRI. Studies were first screened by title and abstract, followed by a full-text review after the first screening. The presence of overlapping patients between potentially eligible studies was also verified.

### Data extraction and quality assessment

We extracted the following data from each selected study by using a standardized form: (1) study characteristics; (2) patient characteristics; (3) unit for analysis; (4) MRI characteristics; (5) image analysis method; (6) reference standard for EPE of PCa; (7) interreader agreement (*κ*) for the binary classification (presence or absence) of each imaging feature; and (8) study outcomes. Some studies evaluated more than one MRI feature, in which case the diagnostic performance of each feature was extracted separately. Details of data extraction and quality assessment are described in the Additional file 1: Methods. The methodological quality of the included studies was evaluated using the Quality Assessment of Diagnostic Accuracy Studies-2 (QUADAS-2) tool [22].

### Data synthesis and statistical analysis

The primary outcome was the diagnostic odds ratio (DOR), which is the ratio between the odds of a test (i.e., each imaging feature) being positive if the subject has a disease (i.e., EPE of PCa) and the odds of the test being positive if the subject does not have the disease. A bivariate random-effects model was employed to determine the meta-analytic pooled DOR and its 95% confidence interval (CI) for each imaging feature. The pooled sensitivity, specificity, and positive and negative likelihood ratios (LRs) and their 95% CIs were obtained for each individual imaging feature. Subgroup analysis was conducted for studies that performed per-patient analysis and studies using only a 3.0-T MRI scanner.

Heterogeneity was assessed using the *I*^2^ statistic, with values greater than 50% being considered to indicate substantial heterogeneity. The presence of a threshold effect was analyzed by the visual assessment of the coupled forest plots of sensitivity and specificity, as well as by calculating the Spearman correlation coefficient between the sensitivity and false-positive rate (a correlation coefficient of 0.6 or higher was considered indicative of a substantial threshold effect) [23]. A meta-regression analysis was conducted to identify factors contributing to substantial heterogeneity, if present. The following covariates were considered for the meta-regression: (1) study design (prospective vs. retrospective), (2) unit for analysis (per patient vs. per lobe), (3) magnetic field strength (3.0-T vs. 1.5-T), (4) use of endorectal coil (yes vs. no), (5) use of anti-peristaltic agent (yes vs. unclear), (6) MRI sequence (multiparametric MRI (mpMRI) [T2-weighted imaging, diffusion-weighted imaging, and dynamic contrast-enhanced imaging] vs. T2-weighted imaging (T2WI) and T1-weighted imaging (T1WI)), (vii) number of MRI readers (single vs. multiple), and (viii) clarity of blinding to reference standard diagnosis (blinded vs. unclear).

To identify outlier studies, residuals of standardized posterior means of random effects and Cook’s distance were used, and then sensitivity analysis was performed after excluding outlier studies. Publication/reporting bias was assessed using Deeks’ funnel plot and Deeks’ asymmetry test. Stata version 16.0 (StataCorp LP, College Station, TX) was used for statistical analysis, with *p* < 0.05 considered statistically significant.

## Results

### Study selection and characteristics

**Fig. 1:**
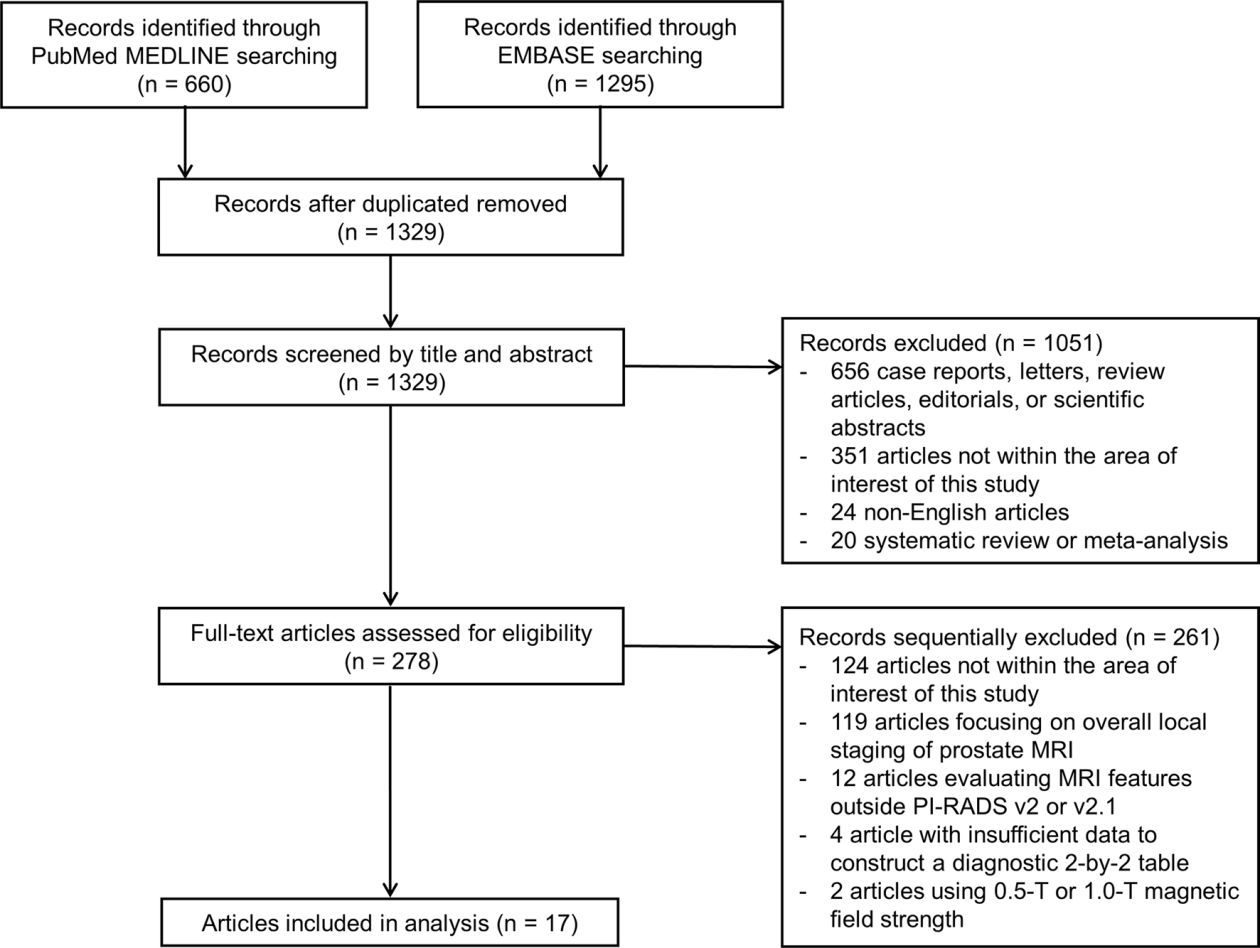
Flow diagram of the study selection process

Of the 1955 studies identified by the initial search, 626 were excluded because of duplication between the PubMed/MEDLINE and EMBASE databases. A total of 1051 studies were then excluded based on a review of the titles and abstracts. As a result of the full-text review, an additional 261 studies were further excluded, and the remaining 17 studies were finally included in this meta-analysis [15, 16, 24–38]. No overlapping populations were identified between the included studies. The study selection process is summarized in Fig. 1.

Table 1 provides a summary of the characteristics of the individual studies. All 17 studies were cohort studies, of which 3 were prospective [15, 27, 28]. The meta-analysis included 3062 patients with a total of 3325 observations. The units of analysis were per patient in 15 studies [15, 16, 24–28, 30–35, 37, 38] and per lobe in two studies [29, 36]. In 11 studies [15, 16, 24, 26, 28, 30, 31, 33, 35–37], MRI was performed using only a 3.0-T scanner. When performing MRI, five studies used an endorectal coil [15, 27, 31, 33, 38], and nine used an anti-peristaltic agent [16, 24, 27, 28, 30, 32, 35, 37, 38].

**Table 1:** Characteristics of included articles

Author (Ref.)	Study design	Period of recruitment	Nation	No. of patient	No. of patient with EPE (%)	Patient age, years*	PSA, ng/mL*	Unit for analysis	MRI magnet	Endorectal coil	Anti-peristaltic agent	MRI sequence^†^	No. of readers (year of experience)	Clarity of blinding review
Ahn H [24]	Retrospective	2011–2016	Korea	221	69 (31.2)	67.2 (range, 45–78)	16.7 ± 17.4	Per patient	3.0-T	No	Yes	mpMRI (T2WI)	2 (19, 3)	Blinded
Alessi S [25]	Retrospective	2012–2013	Italy	301	119 (39.5)	63.15 ± 6.96	8.66 ± 7.94	Per patient	1.5-T	No	NA	mpMRI (NA)	2 (3, 2)	Blinded
Asfuroğlu U [26]	Retrospective	2016–2019	Turkey	79	33 (41.8)	64.5 ± 6.2	9.9 ± 7.4	Per patient	3.0-T	No	NA	mpMRI (T2WI)	2 (10, 4)	Blinded
Beyersdorff D [27]	Prospective	NA	Germany	22	5 (22.7)	62 (range, 50–72)	7.5 (range, 2–14)	Per patient	3.0-T and 1.5-T	No (3.0-T) Yes (1.5-T)	Yes	T2WI, T1WI (NA)	2 (NA)	Blinded
Boesen L [28]	Prospective	2011–2013	Denmark	87	31 (35.6)	65 (range, 47–74)	11 (range, 4.6–45)	Per patient	3.0-T	No	Yes	mpMRI (T2WI)	2 (2, NA)	Blinded
Chen Y [29]	Retrospective	2009–2015	China	353	196 (55.5)	65.9	15.0	Per lobe	3.0-T or 1.5-T	No	NA	mpMRI (NA)	2 (NA)	Unclear
Christophe C [30]	Retrospective	2015–2018	France	92	38 (41.3)	63 (59–67)	PSA density 0.24 (0.15‒0.32)	Per patient	3.0-T	No	Yes	mpMRI (T2WI)	3 (15, 1.5, 1)	Blinded
Costa DN [31]	Retrospective	2015–2016	USA	80	40 (50)	64 ± 8	8.0 ± 6.1	Per patient	3.0-T	Yes	NA	mpMRI (T2WI, ADC map)	5 (NA)	Blinded
Gatti M [32]	Retrospective	2015– 2020	Italy	276	122 (44.2)	Site1: 66 (60–71), Site 2: 67 (62–70)	Site1: 7 (5–10), Site 2: 7 (6–10)	Per patient	1.5-T	No	Yes	mpMRI (NA)	4 (≥ 500 cases analyzed)	Blinded
Gaunay GS [33]	Retrospective	2012–2014	USA	74	24 (32.4)	NA	NA	Per patient	3.0-T	Yes	NA	mpMRI (NA)	1 (fellowship trained radiologist)	Unclear
Matsuoka Y [34]	Retrospective	2007–2015	Japan	210	56 (26.7)	67 (range, 50–81)	7.0 (range, 2.9–30.0)	Per patient	1.5-T	No	NA	mpMRI (T2WI)	2 (10, 5)	Blinded
Mehralivand S [15]	Prospective	2007–2017	USA	553	125 (22.6)	60 ± 8	6.28 (range, 0.21–170)	Per patient	3.0-T	Yes	NA	mpMRI (NA)	1 (15, 9)	Blinded
Onay A [35]	Retrospective	2012–2017	Turkey	110	26 (23.6)	62.7 (range, 40–77)	7.4 (range, 2.1–40)	Per patient	3.0-T	No	Yes	mpMRI (T2WI)	2 (12, 5)	Blinded
Park KJ [16]	Retrospective	2016–2017	Korea	301	129 (42.9)	65 ± 7	7.55 ± 5.62	Per patient	3.0-T	No	Yes	mpMRI (T2WI, DWI, DCE)	2 (> 15, 3)	Blinded
Rosenkrantz AB [36]	Retrospective	NA	USA	90	40 (44.4)	64 ± 8	9.0 ± 11.4	Per lobe	3.0-T	No	NA	mpMRI (T2WI)	2 (4,1)	Blinded
Valentin B [37]	Retrospective	2016–2017	Germany	136	60 (44.1)	67 (62–72)	9.3 (7.0–14)	Per patient	3.0-T	No	Yes	mpMRI (NA)	3 (10, 5, 2)	Unclear
Yu KK [38]	Retrospective	1992–1995	USA	77	34 (44.2)	62.6 ± 7.6	10.7 ± 1.7	Per patient	1.5-T	Yes	Yes	T1WI, T2WI (NA)	3 (≥ 6 months)	Blind

Articles are listed in alphabetical order of the names of the first authors

*EPE*, extraprostatic extension; *PSA*, prostate-specific antigen; *MRI*, magnetic resonance imaging; *mpMRI*, multiparametric MRI; *T2WI*, T2-weighted imaging; *NA*, not available; *T1WI*, T1-weighted imaging; *ADC*, Apparent diffusion coefficient; *DWI*, diffusion-weighted imaging; *DCE*, dynamic contrast-enhanced imaging

*Unless otherwise specified, data are mean or median value, with standard deviation or interquartile range in parenthesis

^†^The parentheses are MRI sequences for evaluating EPE features

### Per-feature diagnosis of EPE of prostate cancer

Of the 17 eligible articles, six reported the diagnostic performance of bulging prostatic contour [15, 28, 30, 32, 33, 38], nine reported that of irregular or spiculated margin [16, 25, 28–32, 34, 36], seven reported that of asymmetry or invasion of the neurovascular bundle [15, 27, 28, 32, 35, 37, 38], five reported that of obliteration of the rectoprostatic angle [15, 27, 32, 35, 38], eight reported that of tumor-capsule interface > 10 mm [16, 24, 26, 30–32, 34, 37], and nine reported that of breach of the capsule with direct tumor extension [15, 16, 28, 29, 31, 32, 34, 35, 37].

**Fig. 2:**
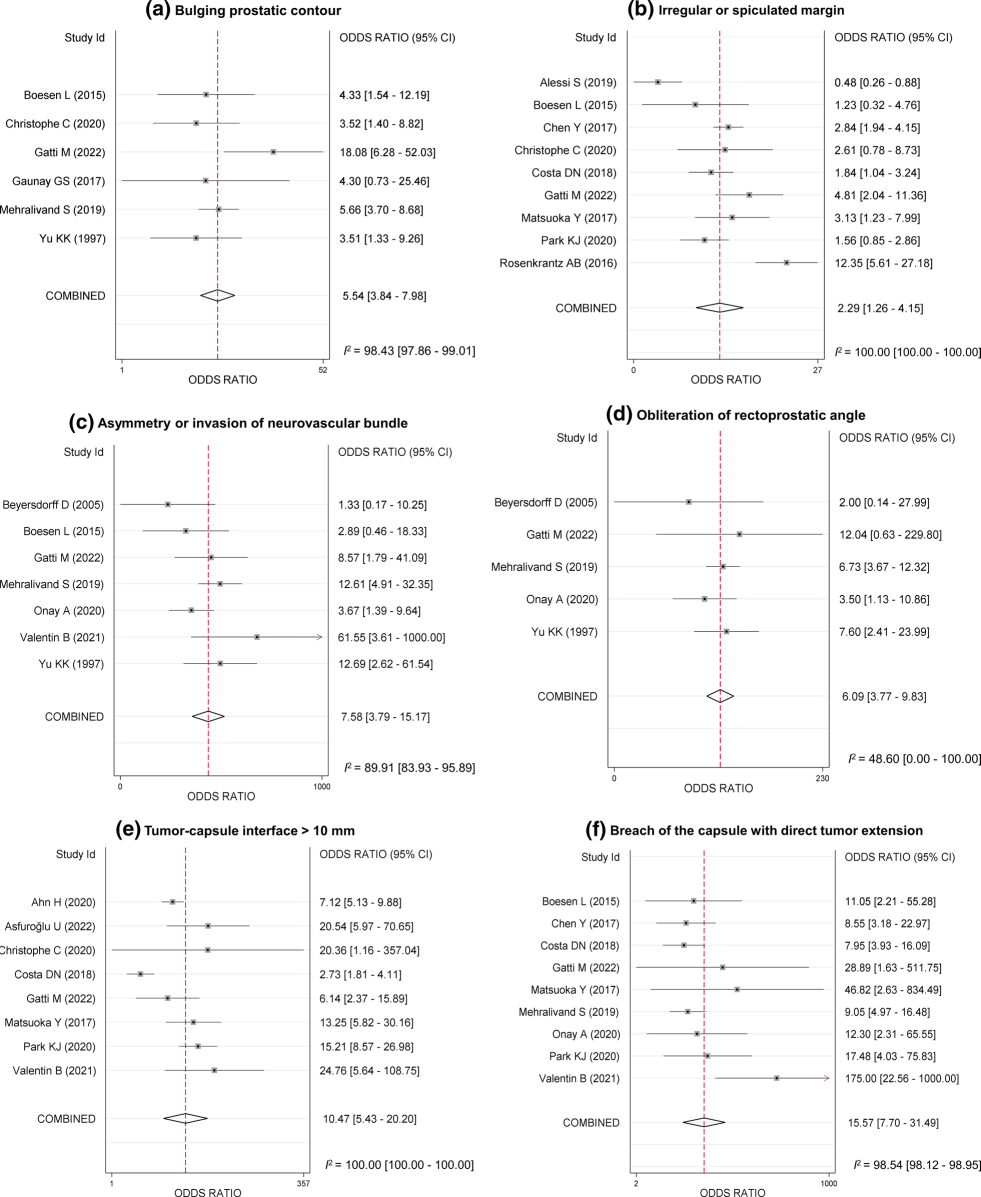
Forest plots of the diagnostic odds ratios of (**a**) bulging prostatic contour, (**b**) irregular or spiculated margin, (**c**) asymmetry or invasion of the neurovascular bundle, (**d**) obliteration of the rectoprostatic angle, (**e**) tumor-capsule interface > 10 mm, and (**f**) breach of the capsule with evidence of direct tumor extension for EPE of prostate cancer

Table 2 and Fig. 2 provide a summary of the pooled DORs of the imaging features for the diagnosis of EPE of PCa. All six imaging features were significantly associated with EPE, with the 95% CIs of the meta-analytic pooled DORs not encompassing 1.0. Of the six features, breach of the capsule with direct tumor extension demonstrated the highest pooled DOR (15.6, 95% CI [7.7–31.5]) followed by tumor-capsule interface > 10 mm (10.5, 95% CI [5.4–20.2]), asymmetry or invasion of the neurovascular bundle (7.6, 95% CI [3.8–15.2]), and obliteration of the rectoprostatic angle (6.1, 95% CI [3.8–9.8]). Irregular or spiculated margin showed the lowest pooled DOR (2.3, 95% CI [1.3–4.2]). There was substantial heterogeneity among the studies in the pooled data except for studies on obliteration of the rectoprostatic angle (*I*^2^, 48.6%). No significant publication bias was noted for all six imaging features (*p* ≥ 0.09; Additional file 1: Fig. S1). The results of subgroup analysis for studies that performed per-patient analysis and those using only a 3.0-T MRI scanner are provided in Additional file 1: Table S1.

**Table 2:** Meta-analytic pooled diagnostic odds ratio of the individual MRI features of PI-RADS for extraprostatic extension

MRI feature	Number of studies	Number of observations	Summary estimates		*P* for publication bias
			Pooled DOR (95% CI)	*I*^2^%	
Bulging prostatic contour	6	978	5.5 (3.8–8.0)	98.4	0.81
Irregular or spiculated margin	9	2377	2.3 (1.3–4.2)	100.0	0.64
Asymmetry or invasion of neurovascular bundle	7	1086	7.6 (3.8–15.2)	89.9	0.32
Obliteration of rectoprostatic angle	5	863	6.1 (3.8–9.8)	48.6	0.55
Tumor-capsule interface > 10 mm	8	2202	10.5 (5.4–20.2)	100.0	0.16
Breach of the capsule with direct tumor extension	9	2603	15.6 (7.7–31.5)	98.5	0.09

*MRI*, magnetic resonance imaging; *PI-RADS*, prostate imaging reporting and data system; *DOR*, diagnostic odds ratio; *CI*, confidence interval

The pooled sensitivities and specificities of the six imaging features are summarized in Table 3 and Additional file 1: Fig. S2. Breach of the capsule with direct tumor extension showed the highest pooled specificity of 98.0% (95% CI, 96.2–99.0), followed by asymmetry or invasion of the neurovascular bundle (95.1%, 95% CI [87.9–98.1]) and obliteration of the rectoprostatic angle (94.5%, 95% CI [88.6–97.4]). However, the pooled sensitivity of these imaging features was fairly low, ranging from 23.7 to 28.0%. In contrast, tumor-capsule interface > 10 mm showed the highest pooled sensitivity of 86.3% (95% CI, 70.0–94.4), although the pooled specificity was modest (62.5%, 95% CI [47.3–75.6]). Among the six imaging features, bulging prostatic contour, asymmetry or invasion of the neurovascular bundle, obliteration of the rectoprostatic angle, and breach of the capsule with evidence of direct tumor extension exhibited substantial threshold effects (Spearman correlation coefficient ≥ 0.66).

**Table 3:** Meta-analytic pooled indices of diagnostic test accuracy for the individual MRI features

MRI feature	No. of studies	Summary estimates			
		Sensitivity % (95% CI), *I*^2^%	Specificity % (95% CI), *I*^2^%	PLR (95% CI)	NLR (95% CI)
Bulging prostatic contour	6	53.0 (36.6–68.7), 82.2	83.1 (71.6–90.6), 81.5	3.1 (2.2–4.4)	0.6 (0.4–0.7)
Irregular or spiculated margin	9	28.4 (16.3–44.7), 93.7	85.2 (79.3–89.6), 85.4	1.9 (1.2–3.0)	0.8 (0.7–1.0)
Asymmetry or invasion of neurovascular bundle	7	28.0 (19.0–39.3), 75.5	95.1 (87.9–98.1), 92.3	5.7 (2.8–11.6)	0.8 (0.7–0.8)
Obliteration of rectoprostatic angle	5	26.3 (14.8–42.2), 77.0	94.5 (88.6–97.4), 61.7	4.8 (3.0–7.5)	0.8 (0.7–0.9)
Tumor-capsule interface > 10 mm	8	86.3 (70.0–94.4), 87.7	62.5 (47.3–75.6), 92.6	2.3 (1.7–3.1)	0.2 (0.1–0.4)
Breach of the capsule with direct tumor extension	9	23.7 (14.8–35.8), 93.0	98.0 (96.2–99.0), 78.1	12.1 (6.4–23.0)	0.8 (0.7–0.9)

*MRI*, magnetic resonance imaging; *CI*, confidence interval; *PLR*, positive likelihood ratio; *LNR*, negative likelihood ratio

### Meta-regression analysis

Meta-regression analysis (Additional file 1: Table S2) showed that the study design, magnetic field strength, use of an endorectal coil, and clarity of blinding review were significant contributing factors to study heterogeneity. Prospective studies showed a lower sensitivity for asymmetry or invasion of the neurovascular bundle (17% vs. 33%) than retrospective studies. Studies using only 3.0-T MRI showed a higher sensitivity for breach of the capsule with evidence of direct tumor extension (32% vs. 12%) than other studies. Studies using endorectal coils showed lower specificities for tumor-capsule interface > 10 mm (55% vs. 63%) and breach of the capsule with evidence of direct tumor extension (95% vs. 99%) than studies that did not use these coils. Studies that performed blinded reviews tended to show lower specificities for bulging prostatic contour (79% vs. 96%) and asymmetry or invasion of the neurovascular bundle (94% vs. 100%) than studies that were unclear.

### Sensitivity analysis

One study each for irregular or spiculated margin [32], tumor-capsule interface > 10 mm [30], and breach of the capsule with direct tumor extension [37] was identified as an outlier study showing a standardized residual of > ± 2 (Additional file 1: Fig. S3). After excluding the outlier studies, the pooled DOR, sensitivity, and specificity of the imaging features were similar to those before exclusion (Additional file 1: Table S3). No outlier studies were identified for bulging prostatic contour, asymmetry or invasion of the neurovascular bundle, and obliteration of the rectoprostatic angle.

### Interreader agreement

Four studies reported interreader agreements for a binary classification of tumor-capsule interface > 10 mm [24, 26, 30, 37], which were moderate to substantial (*κ*, 0.43–0.75). The interreader agreement for bulging prostatic contour was reported to be moderate (*κ*, 0.59) in one study [32], that for irregular or spiculated margin was moderate (*κ*, 0.59) in two studies [32, 36], and that for asymmetry or invasion of the neurovascular bundle was fair (*κ*, 0.34) in one study [32]. The interreader agreement for breach of the capsule with evidence of direct tumor extension was almost perfect (*κ*, 0.84) in one study [32]. Because the interreader agreement was reported in a small number of studies, we performed only a qualitative synthesis.

### Quality assessment

**Fig. 3:**
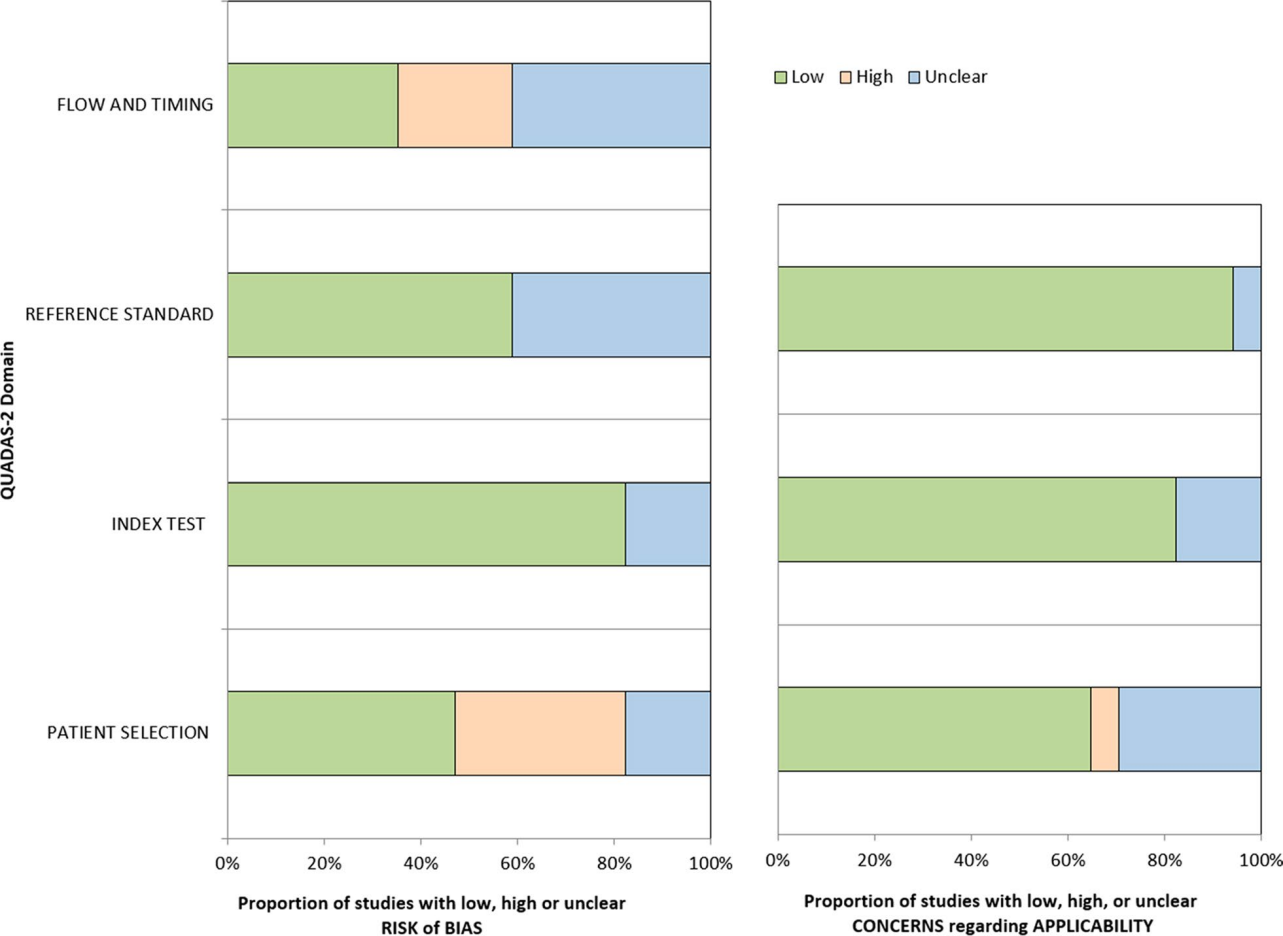
QUADAS-2 assessment. The methodological quality distribution of the articles is presented as the proportions of articles (0–100%) regarding risk of bias and concerns of applicability (low, high, or unclear) for each domain

The quality assessment is summarized in Fig. 3. In the patient selection domain, nine studies were judged to be at high or unclear risk of bias due to the retrospective design and ambiguity on whether they avoided inappropriate exclusions [24, 26, 29, 30, 33, 34, 36–38]. In the flow and timing domain, four studies had a high risk of bias due to an inappropriate interval (> 3 months) between the index test and reference standard [26, 31, 32, 35]. In the reference standard domain, seven studies had an unclear risk of bias because they did not clearly explain how histopathological EPE was determined [33] or whether the reference standard diagnosis of EPE was interpreted without knowledge of the results of the index test [16, 32–37]. In the index test domain, three studies had an unclear risk of bias because they did not explicitly state whether the interpretation of the index test was blinded to the reference standard [29, 33, 37].

## Discussion

The current meta-analysis assessed the diagnostic performance of the six EPE-related MRI features in PI-RADS versions 2 and 2.1. Despite varying degrees of sensitivity and specificity, all image features showed significant DORs to predict EPE. The two findings with the greatest DORs in order were breach of the capsule with direct tumor extension and tumor-capsule interface > 10 mm. The DOR was lowest for the irregular or spiculated margin feature. Based on the various DORs of each finding in the current study, the removal of the scoring system for EPE with rather arbitrary scores from PI-RADS 1 was plausible. Radiologists should comprehend each feature’s diagnostic performance and evaluate EPE using appropriate features that are suitable for the purpose of the examination.

In this meta-analysis, breach of the capsule with direct tumor extension showed the greatest DOR (15.6) with the highest pooled specificity (98.0%) and the lowest pooled sensitivity (23.7%) among the six MRI findings. A similar pattern was seen for asymmetry or invasion of the neurovascular bundle and obliteration of the rectoprostatic angle (pooled DOR, 6.1–7.6; pooled sensitivity, 26.3–28.0%; pooled specificity, 94.5–95.1%). The resulting high positive LRs of these features are generally in line with the previous ESUR or EPE grading system [14, 15], which assigns high scores to these features. However, in return, the negative LRs of these features were large (0.8), indicating that they are not useful to rule out EPE. These three findings, which describe the existence of tumor signal intensity outside of the prostate gland, may lead to focal/microscopic EPE with a few tumor cells beyond the prostate gland being missed [39]. Overall, the presence of these three MRI features (breach of the capsule with direct tumor extension, asymmetry or invasion of the neurovascular bundle and obliteration of the rectoprostatic angle) increases radiologists’ confidence in predicting positive EPE, but the absence of these features does not preclude EPE.

The length of the tumor-capsule interface is an indirect sign of EPE, implying that the longer the interface is, the greater the likelihood of EPE. Tumor-capsule interface > 10 mm showed the highest pooled sensitivity (86.3%) and the lowest pooled specificity (62.5%) and negative LR (0.2) for predicting EPE. Some studies suggested that a larger threshold, such as 15 mm, should be used to predict EPE because a 10 mm threshold may result in an excessive number of false-positive cases [40–42]. The lowest pooled specificity in the current meta-analysis is consistent with concerns about a high number of false-positive cases. A lower threshold, however, was proposed in a study to identify focal EPE [36]. Although several thresholds have been proposed, a 10 mm cutoff is used in PI-RADS v2. Tumor-capsule interface > 10 mm, which was the only MRI finding with relatively high sensitivity in this meta-analysis, would be important in detecting focal EPE. When it is critical to rule in even the slim possibility of EPE, such as when performing local treatment or nerve-sparing surgery, radiologists should evaluate EPE based on the most sensitive feature.

Substantial threshold effects were seen for bulging prostatic contour, asymmetry or invasion of the neurovascular bundle, obliteration of the rectoprostatic angle, and breach of the capsule with evidence of direct tumor extension (Spearman correlation coefficient ≥ 0.66), suggesting that authors used different thresholds or criteria to determine a positive test result [23]. The MRI features are not qualitatively defined, making them subjective. Although there are several pictorial reviews on how to interpret PI-RADS, EPE-related MRI findings have rarely been depicted [43–45]. Because lesions with EPE are classified as PI-RADS 5, more precise descriptions with more explicit MRI examples for each feature may assist in improving the interreader agreement between image readers as well as the tumor staging accuracy.

This meta-analysis has several limitations. First, the heterogeneity across the included studies potentially limits the generalization of meta-analytic summary estimates. To address this issue, subgroup meta-regression and sensitivity analyses were performed to determine the cause of the heterogeneity in our study, but it still remains a matter of concern. Second, most studies were retrospective (82.4%, 14/17), and the number of included studies for each imaging feature was small. Third, we analyzed the MRI findings to predict EPE (T3a) that were mentioned in PI-RADS version 2, but we excluded findings to indicate seminal vesicle invasion, which are for a distinct T stage of a tumor (T3b). Furthermore, we did not evaluate other EPE evaluation systems (e.g., EPE grading system) or EPE-suggestive features outside PI-RADS (e.g., length of tumor capsular contact > 15 mm) since more validation seems necessary for the findings.

In conclusion, our meta-analysis provided a systematic and comprehensive summary of individual MRI features according to PI-RADS to predict EPE in PCa. Among the six MRI features of PCa, breach of the capsule with direct tumor extension and tumor-capsule interface > 10 mm were the most predictive of EPE with the highest specificity and sensitivity, respectively. These results could be helpful for risk classification and more evidence-based standardized reviews to evaluate the EPE of PCa.

## Supplementary Information

**Additional file 1.** Supplementary material.

## Data Availability

All data generated or analyzed during this study are included in this published article and its Additional files.

## Abbreviations

CI: Confidence interval
DOR: Diagnostic odds ratio
EPE: Extraprostatic extension
LR: Likelihood ratio
mpMRI: Multiparametric MRI
MRI: Magnetic resonance imaging
PCa: Prostate cancer
PI-RADS: Prostate imaging-reporting and data system
PSA: Prostate-specific antigen
QUADAS: Quality assessment of diagnostic accuracy studies
RP: Radical prostatectomy
