# Supplementary material for: Imaging features of the PI-RADS for predicting extraprostatic extension of prostate cancer: systematic review and meta-analysis
Source: Insights Imaging. 2023 May 8;14:77. doi: 10.1186/s13244-023-01422-9 (PMC10167060; doi:10.1186/s13244-023-01422-9)
Supplement: Supplementary file 1 — Additional file 1. Supplementary material. [file 13244_2023_1422_MOESM1_ESM.pdf]

Imaging features of the PI-RADS for predicting extraprostatic extension of prostate cancer: Systematic review and meta-analysis

Supplementary Methods

Data extraction and quality assessment

We extracted the following data from each selected study by using a standardized form: (1) study characteristics, including first author, year and nation of publication, period of patient recruitment, and study design (prospective or retrospective cohort study or case–control study); (2) patient characteristics, including number of patients, age, and serum prostate-specific antigen (PSA) level; (3) unit for analysis (per patient or per lobe analysis); (4) MRI characteristics, including magnetic field strength (1.5-T or 3.0-T), use of an endorectal coil (endorectal or pelvic phased array), use of an anti-peristaltic agent, and detailed MRI sequence; (5) image analysis method, including number of image readers, experience level of each reader in prostate imaging, and clarity of blinding to reference standards when conducting reviews; (6) reference standard for EPE of prostate cancer; (7) interreader agreement (κ) for the binary classification (presence or absence) of each imaging feature; and (8) study outcomes, including true-positive, false-positive, false-negative, and true-negative values of each imaging feature associated with EPE. When not reported explicitly, data were extracted manually from the text, tables, and figures. For studies with more than one image reader, i.e., multiple index test readers, the dataset with the highest accuracy (Youden's index) was selected for the meta-analysis.

The methodological quality of the included studies was evaluated using the Quality Assessment of Diagnostic Accuracy Studies-2 (QUADAS-2) tool. All included studies were assessed in the patient selection, index test, and reference standard domains in terms of risk of bias and concerns of applicability, as well as in the flow and timing domain in terms of risk of bias.

Supplementary Table 1. Subgroup analysis for studies that performed per-patient analysis and studies using only a 3.0-T MRI.

MRI feature	*Pooled DOR (95% CI)*		
	All included studies	*Per-patient analysis*	*3.0-T MRI*
Bulging prostatic contour	5.5 (3.8–8.0)	5.5 (3.8–8.0)	4.3 (2.5–7.5)
Irregular or spiculated margin	2.3 (1.3–4.2)	1.7 (1.0–3.0)	2.4 (1.1–5.3)
Asymmetry or invasion of neurovascular bundle	7.6 (3.8–15.2)	7.6 (3.8–15.2)	9.5 (3.6–25.0)
Obliteration of rectoprostatic angle	6.1 (3.8–9.8)	6.1 (3.8–9.8)	NA*
Tumor-capsule interface > 10 mm	10.5 (5.4–20.2)	10.5 (5.4–20.2)	11.6 (4.7–28.5)
Breach of the capsule with direct tumor extension	15.6 (7.7–31.5)	18.0 (7.8–41.8)	13.6 (5.9–31.5)

* Meta-analytic pooling is not applicable due to the small number of studies (n = 2).

MRI, magnetic resonance imaging; DOR, diagnostic odds ratio; CI, confidence interval; NA, not applicable.

Supplementary Table 2. Meta-regression analysis to identify sources of heterogeneity for imaging features in prediction of extraprostatic extension.

Covariates	*Number of studies*	*Meta-analytic summary estimates*		
		Sensitivity (95% CI)	*Specificity (95% CI)*	*P*
Bulging prostatic contour				
Study design				0.98
Prospective	2	53% (25, 81)	83% (67, 98)	
Retrospective	4	53% (32, 74)	84% (72, 95)	
Magnetic field strength				0.11
3.0-T	4	44% (29, 60)	87% (79, 96)	
1.5-T	2	73% (55, 91)	75% (56, 94)	
Use of endorectal coil				0.67
Yes	3	53% (28, 77)	81% (68, 94)	
No	3	54% (29, 78)	85% (74, 96)	
Use of antiperistaltic agent				0.50
Yes	4	60% (41, 78)	79% (67, 92)	
Unclear	2	40% (14, 66)	89% (78, 100)	
MRI sequence				0.08
mpMRI	5	47% (32, 63)	85% (78, 91)	
T2WI and T1WI	1	74% (46, 100)	56% (31, 81)	
Number of MRI readers				0.50
Single	2	40% (14, 66)	89% (78, 100)	
Multiple	4	60% (41, 78)	79% (67, 92)	
Clarity of blinding review				0.03
Blinded	5	60% (51, 69)	79% (71, 87)	
Unclear	1	16% (0, 34)	96% (89, 100)	
Irregular or spiculated margin				
Study design				0.60
Prospective	1	12% (0, 36)	90% (77, 100)	
Retrospective	8	31% (16, 46)	85% (79, 90)	
Unit for analysis				0.17
Per patient	7	23% (10, 35)	85% (79, 91)	
Per lobe	2	52% (20, 84)	84% (73, 95)	
Magnetic field strength				0.49
3.0-T	5	26% (8, 45)	88% (82, 93)	
1.5-T/ 1.5-T or 3.0-T	4	31% (9, 54)	82% (74, 90)	
Use of endorectal coil				0.78
Yes	1	18% (0, 49)	89% (78, 100)	

No	8	30% (14, 46)	85% (79, 90)	
Use of anti-peristatic agent				0.94
Yes	4	29% (7, 51)	84% (76, 93)	
Unclear	5	28% (9, 47)	86% (80, 92)	
Clarity of blinding review				0.94
Blinded	8	28% (13, 43)	85% (79, 90)	
Unclear	1	31% (0, 75)	86% (73, 99)	
Asymmetry or invasion of neurovascular bundle				
Study design				0.04
Prospective	3	17% (9, 26)	95% (86, 100)	
Retrospective	4	33% (24, 42)	96% (91, 100)	
Magnetic field strength				0.67
3.0-T	5	27% (15, 38)	95% (89, 100)	
1.5-T	2	31% (12, 49)	96% (90, 100)	
Use of endorectal coil				0.66
Yes	2	23% (8, 38)	97% (93, 100)	
No	5	30% (17, 43)	93% (87, 100)	
Use of antiperistatic agent				0.25
Yes	6	31% (22, 40)	94% (88, 100)	
Unclear	1	15% (5, 25)	99% (96, 100)	
MRI sequence				0.17
mpMRI	5	23% (15, 31)	97% (94, 100)	
T2WI and T1WI	2	44% (22, 66)	86% (68, 100)	
Number of MRI readers				0.25
Single	1	15% (5, 25)	99% (96, 100)	
Multiple	6	31% (22, 40)	94% (88, 100)	
Clarity of blinding review				0.04
Blinded	6	28% (17, 39)	94% (88, 99)	
Unclear	1	28% (4, 52)	100% (100, 100)	
Obliteration of rectoprostatic angle				
Study design				0.97
Prospective	2	27% (4, 50)	95% (88, 100)	
Retrospective	3	26% (8, 43)	94% (88, 100)	
Magnetic field strength				0.65
3.0-T	3	28% (10, 46)	93% (86, 100)	
1.5-T	2	25% (4, 45)	97% (91, 100)	
Use of endorectal coil				0.28
Yes	2	35% (16, 55)	93% (86, 99)	
No	3	17% (4, 30)	95% (90, 100)	

Use of antiperistaltic agent				0.82
Yes	4	26% (9, 42)	93% (88, 99)	
Unclear	1	25% (0, 51)	95% (90, 100)	
MRI sequence				0.24
mpMRI	3	20% (11, 30)	96% (93, 99)	
T2WI and T1WI	2	44% (21, 66)	90% (79, 100)	
Number of MRI readers				0.82
Single	1	25% (0, 51)	95% (90, 100)	
Multiple	4	26% (9, 42)	93% (88, 99)	
Tumor-capsule interface > 10 mm				
Country of origin				0.46
Western	5	88% (75, 100)	56% (38, 73)	
Eastern	3	82% (60, 100)	71% (53, 89)	
Magnetic field strength				0.33
3.0-T	6	89% (79, 100)	57% (41, 72)	
1.5-T	2	72% (37, 100)	78% (60, 97)	
Use of endorectal coil				0.01
Yes	1	70% (15, 100)	55% (13, 96)	
No	7	88% (77, 99)	63% (48, 78)	
Use of antiperistaltic agent				0.70
Yes	5	89% (77, 100)	61% (43, 80)	
Unclear	3	81% (56, 100)	65% (42, 88)	
Clarity of blinding review				0.42
Blinded	7	83% (70, 95)	65% (50, 79)	
Unclear	1	97% (90, 100)	46% (5, 87)	
Breach of the capsule with evidence of direct tumor extension				
Study design				0.25
Prospective	2	29% (4, 54)	96% (92, 99)	
Retrospective	7	22% (11, 34)	98% (97, 99)	
Unit for analysis				0.26
Per patient	8	27% (17, 37)	98% (96, 99)	
Per lobe	1	8% (0, 19)	99% (97, 100)	
Magnetic field strength				0.01
3.0-T	6	32% (20, 44)	97% (95, 98)	
1.5-T/ 1.5-T or 3.0-T	3	12% (4, 21)	99% (99, 100)	
Use of endorectal coil				0.01
Yes	2	29% (5, 54)	95% (93, 97)	
No	7	22% (11, 34)	99% (98, 100)	
Use of anti-peristaltic agent				0.07

Yes	5	31% (15, 46)	99% (97, 100)	
Unclear	4	17% (6, 29)	98% (96, 99)	
Number of MRI readers				0.33
Single	1	29% (0, 64)	96% (91, 100)	
Multiple	8	23% (12, 34)	98% (97, 99)	
Clarity of blinding review				0.36
Blinded	7	21% (10, 33)	97% (96, 99)	
Unclear	2	31% (6, 56)	98% (97, 100)	

CI, confidence interval; MRI, magnetic resonance imaging; mpMRI, multiparametric MRI; T2WI, T2-weighted imaging; T1WI, T1-weighted imaging.

Supplementary Table 3. Results of sensitivity analysis after excluding outlier studies.

MRI feature	*No. of studies*	*Meta-analytic summary estimates*					
		DOR (95% CI)	I^2 %	*Sensitivity % (95% CI)*	I^2 %	*Specificity % (95% CI)*	I^2 %
Irregular or spiculated margin							
Before outlier study exclusion	9	2.3 (1.3–4.2)	100.0	28.4 (16.3–44.7)	93.7	85.2 (79.3–89.6)	85.4
After outlier study* exclusion	8	2.1 (1.1–3.9)	100.0	23.9 (14.6–36.7)	91.4	86.7 (82.3–90.2)	79.4
Tumor-capsule interface >10 mm							
Before outlier study exclusion	8	10.5 (5.4–20.2)	100.0	86.3 (70.0–94.4)	87.7	62.5 (47.3–75.6)	92.6
After outlier study† exclusion	7	9.1 (5.1–16.1)	100.0	80.9 (68.3–89.3)	86.9	68.2 (58.1–76.7)	87.3
Breach of the capsule with direct tumor extension							
Before outlier study exclusion	9	15.6 (7.7–31.5)	98.5	23.7 (14.8–35.8)	93.0	98.0 (96.2–99.0)	78.1
After outlier study‡ exclusion	8	11.3 (7.2–17.7)	44.1	19.1 (13.6–26.1)	83.6	98.0 (96.2–99.0)	71.7

* Gatti M et al (2022) [32].

† Christophe C et al (2020) [30].

‡ Valentin B et al (2021) [37].

DOR, diagnostic odds ratio; CI, confidence interval.

Supplementary Figure 1. Deeks' funnel plot asymmetry test to evaluate publication bias for the imaging features. (A) bulging prostatic contour, (B) irregular or spiculated margin, (C) asymmetry or invasion of the neurovascular bundle, (D) obliteration of the rectoprostatic angle, (E) tumor-capsule interface >10 mm, and (F) breach of the capsule with evidence of direct tumor extension.

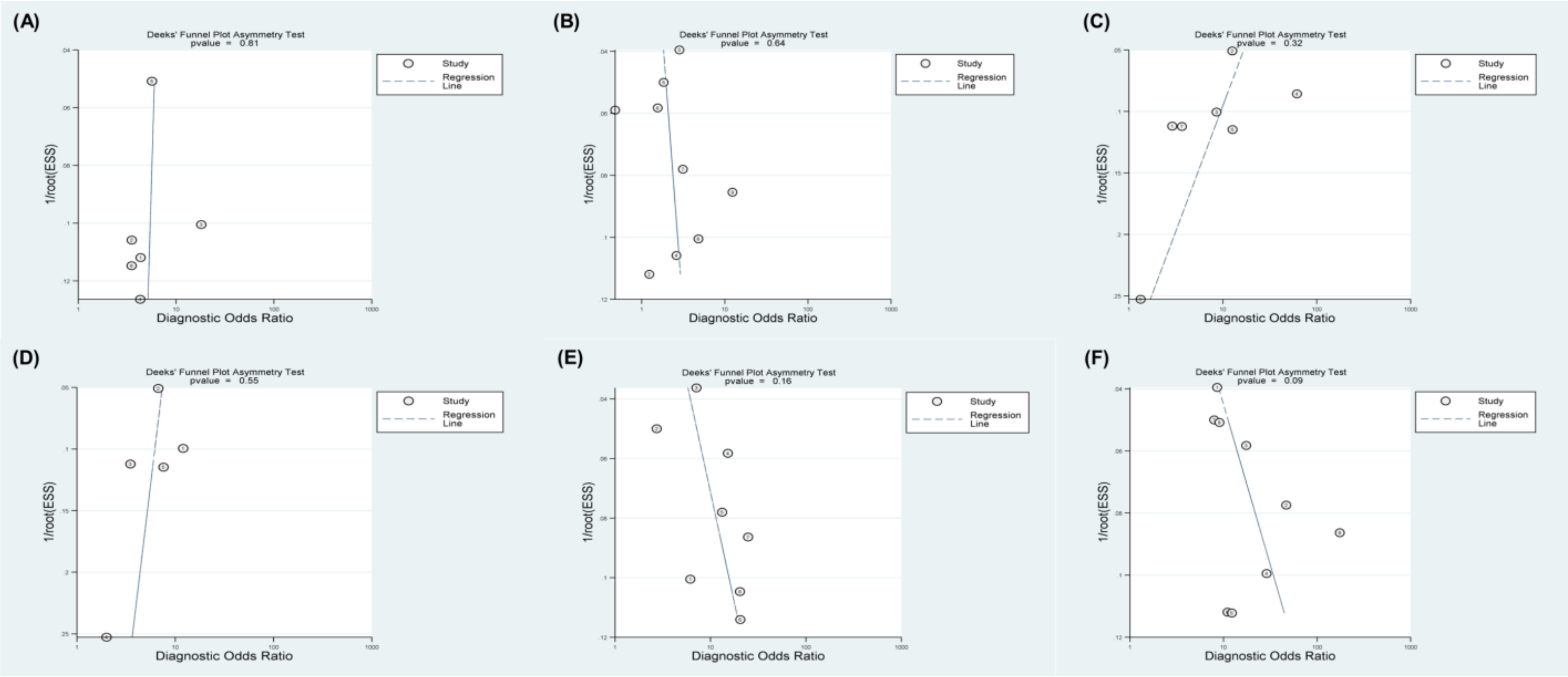

Supplementary Figure 2. Coupled forest plots of sensitivity and specificity for each imaging feature in prediction of extraprostatic extension. (A) bulging prostatic contour, (B) irregular or spiculated margin, (C) asymmetry or invasion of the neurovascular bundle, (D) obliteration of the rectoprostatic angle, (E) tumor-capsule interface >10 mm, and (F) breach of the capsule with evidence of direct tumor extension.

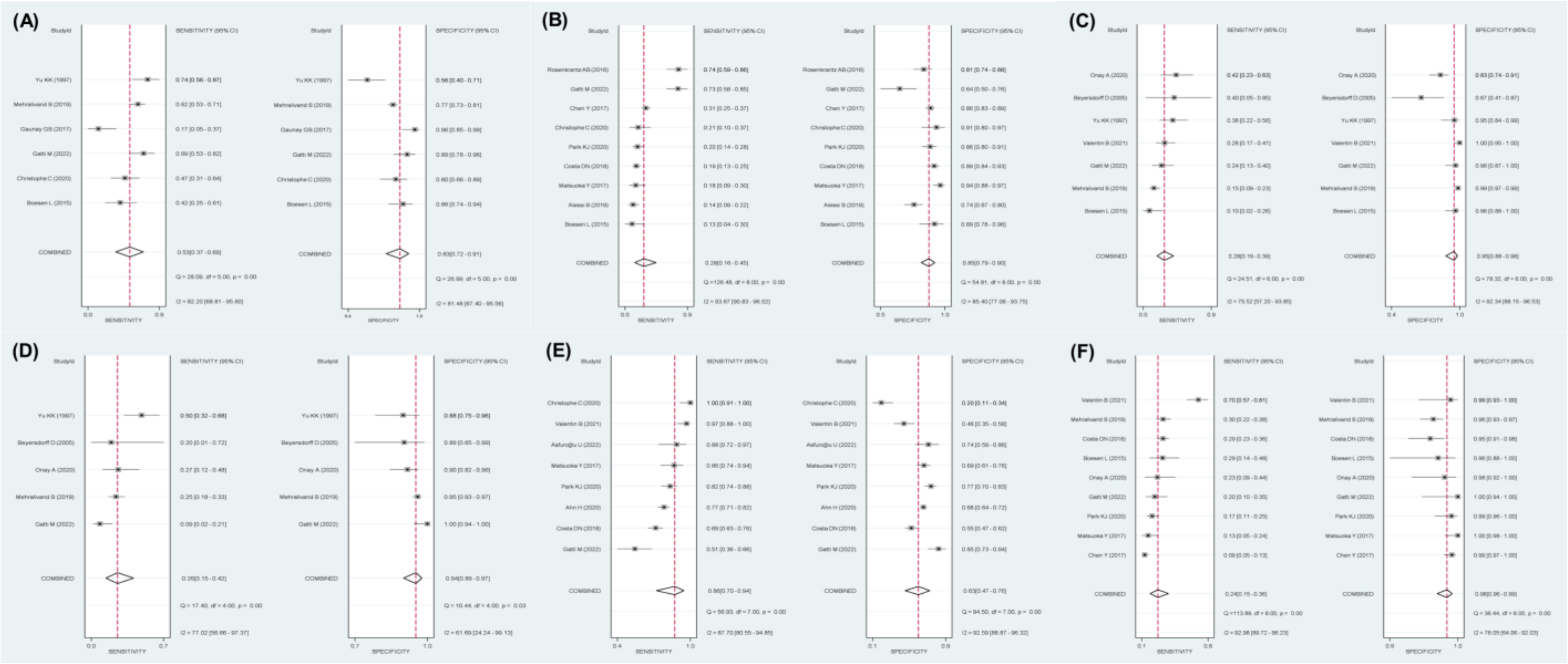

Supplementary Figure 3. Model checking for identifying outlier studies for each imaging feature. (A) bulging prostatic contour, (B) irregular or spiculated margin, (C) asymmetry or invasion of the neurovascular bundle, (D) obliteration of the rectoprostatic angle, (E) tumor-capsule interface >10 mm, and (F) breach of the capsule with evidence of direct tumor extension.

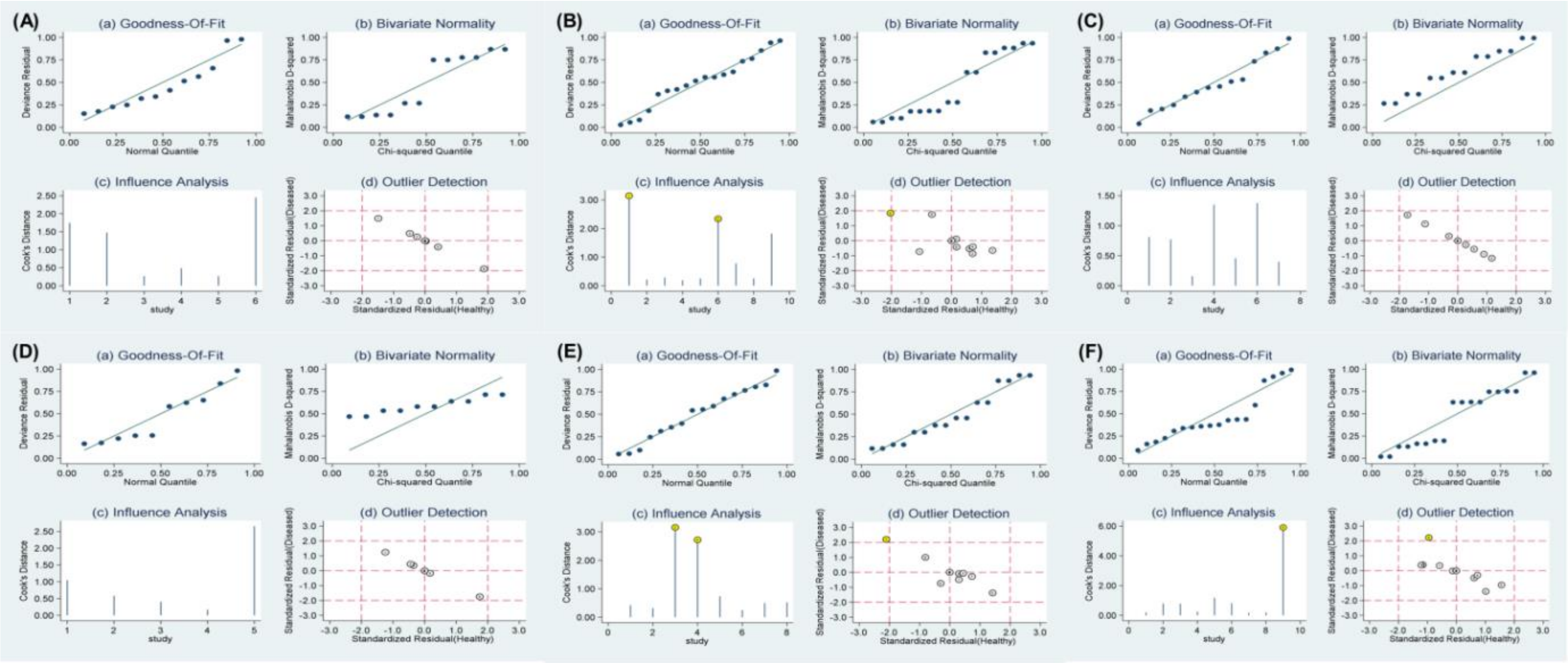

(a) Quantile plot for residual-based goodness-of-fit. (b) Chi-squared probability plot of assessment of bivariate normality assumption. (c) Influence analysis for Cook’s distance. (d) Outlier detection through standardized predicted random effects.
